# Distribución espacial de egresos hospitalarios de casos por infección vírica por picadura de mosquito en México entre 2004 y 2014

**DOI:** 10.26633/RPSP.2017.30

**Published:** 2017-10-12

**Authors:** José Luis Manzanares

**Affiliations:** 1 Colegio de la Frontera Norte Colegio de la Frontera Norte Nogales México Colegio de la Frontera Norte, Nogales, México.

**Keywords:** Geografía médica, dengue, virus Chikungunya, infección por el virus Zika, México, Geography, Medical, dengue, Chikungunya virus, Zika virus infection, Mexico, Geografia médica, dengue, virus Chikungunya, infecção pelo Zika virus

## Abstract

**Objetivo.:**

*Conocer la distribución espacial de los egresos hospitalarios por fiebre vírica por picadura de mosquito (FVPM) en México y caracterizar su evolución histórica en el periodo 2004-2014*.

**Métodos.:**

*Se realizo un análisis exploratorio e inferencial de corte transversal. Los datos primarios se obtuvieron de los registros de egresos hospitalarios del Sistema Nacional de Información en Salud (SINAIS) del periodo 2004-2014. Para identificar clusters de los casos notificados, se estimaron indicadores de asociación geográfica y establecieron zonas de riesgo sobre la base de determinantes ambientales que se validaron con medidas estimadas de concentración relativa. Para conocer la evolución temporal y determinar la estructura de edad de los casos, se estimaron tasas de crecimiento y se construyeron curvas de densidad*.

**Resultados.:**

*La distribución geográfica de estos egresos hospitalarios en México sigue un patrón focalizado. En total se detectó alta concentración en clusters integrados por 20 municipios de los cuales 9 se localizaron en el estado de Sinaloa. En 37% de los estados del país las concentraciones de egresos hospitalarios por FVPM fueron mayores que las del país en conjunto y sus índices de localización oscilaron entre 5,79 en Sinaloa y 1,17 en Campeche*.

**Conclusión.:**

*La información geográfica obtenida, la referente al vínculo encontrado entre las zonas de alto riesgo de transmisión de la infección por la presencia del vector transmisor y la relativa a las condiciones sociales como la pobreza son útiles para diseñar estrategias de prevención y control de las infecciones víricas emergentes transmitidas por artrópodos en México, como la del virus del Zika*.

## Abstract (en)

***Objective*.:**

Determine the spatial distribution of hospital discharges of viral fever caused by mosquito bites in Mexico and characterize its historical trend in the period 2004–2014.

An exploratory cross-sectional inferential analysis was performed. The primary data were obtained from the hospital discharge registries of the National Health Information System (SINAIS) for the period 2004-2014. In order to identify clusters of reported cases, indicators of geographical association were estimated and risk areas established on the basis of environmental determinants validated with estimated relative concentration measurements. In order to determine the temporal trend and age structure of the cases, growth rates were estimated and density curves constructed.

***Results*.:**

The geographic distribution of these hospital discharges in Mexico follows a focalized pattern. In total, a high concentration was detected in clusters made up of 20 municipalities, 9 of which were located in the state of Sinaloa. In 37% of states in the country, the concentrations of hospital discharges of viral fever cases were higher than those for the country as a whole, and their localization indices ranged from 5.79 in Sinaloa to 1.17 in Campeche.

**Conclusion.:**

Geographic information, information on the link found between areas at high risk for transmission of infection due to the presence of the transmitting vector, and information on social conditions such as poverty are useful for designing strategies for the prevention and control of emerging arthropod-borne viral infections, such as the Zika virus, in Mexico.

## RESUMO

**Objetivo.:**

Conhecer a distribuição espacial dos egressos hospitalares de febre viral por picada de mosquitos (FVPM) no México e caracterizar sua evolução histórica no período 2004–2014.

**Métodos.:**

Foi realizado um estudo exploratório e inferencial de corte transversal. Os dados primários foram obtidos dos registros de egressos hospitalares do Sistema Nacional de Informação em Saúde (SINAIS) para o período 2004–2014. Para identificar clusters dos casos notificados, foram estimados indicadores de associação geográfica e estabelecidas as áreas de risco com base em determinantes ambientais que foram validados com medidas estimadas de concentração relativa. Para se conhecer a evolução temporal e determinar a estrutura etária dos casos, foram estimadas taxas de crescimento e construídas curvas de densidade.

**Resultados.:**

A distribuição geográfica dos egressos hospitalares no México segue um padrão focalizado. No todo, verificou-se alta concentração em clusters formados por 20 municípios, dos quais 9 estavam situados no Estado de Sinaloa. Em 37% dos Estados do país, a concentração de egressos hospitalares por FVPM foi maior que a do país como um todo e os índices de localização variaram de 5,79 em Sinaloa a 1,17 em Campeche.

**Conclusão.:**

Os dados geográficos obtidos, relativos à associação verificada entre as áreas de alto risco de transmissão de infecção pela presença dos vetores transmissores e condições sociais como pobreza, são úteis para o planejamento de estratégias de prevenção e controle de infecções virais emergentes transmitidas por artrópodes no México, como a do vírus zika.

Aunque las infecciones víricas transmitidas por picadura de mosquito no son un fenómeno reciente, en la actualidad han adquirido relevancia sobre todo por su acelerada expansión y por su aparente vínculo con manifestaciones neurológicas graves observadas en enfermedades como la producida por virus del Zika, una afección que constituye una emergencia de salud pública internacional y para la cual no se dispone aún de un fármaco antivírico específico (1). En la comunidad científica existe un consenso sobre el vínculo de la infección por este virus con padecimientos como el dengue, la fiebre chikungunya (2-4) o la fiebre amarilla (5) debido a que el vector trasmisor asociado son los mosquitos del género *flavivirus* (6) *Aedes aegypti* y *Aedes albopictus* (7). En México, este último se detectó por primera vez en 1993 en el estado de Coahuila, en la frontera con los Estados Unidos de América (8).

En México, durante 2014 se notificaron 20 682 casos de fiebre vírica por picadura de mosquito (FVPM), incluidos los casos de dengue y de chikungunya. Del total de casos por FVPM, 13 682 se detectaron en las unidades médicas de instituciones públicas como el Instituto Mexicano del Seguro Social (IMSS) y el Instituto de Seguridad y Servicios Sociales de los Trabajadores del Estado (ISSSTE) y 7 000, en las unidades médicas de la Secretaría de Salud y los Servicios Estatales de Salud, donde 71,54% de los casos correspondieron a pacientes cuya adscripción para el cuidado de salud es la modalidad Seguro Popular. Este esquema está dirigido a individuos en situación vulnerable por sus indicadores de pobreza, un factor contextual que amplifica el riesgo para la salud pública que representa la expansión de estos virus.

La importancia que tiene conocer mejor la diseminación geográfica de las enfermedades asociadas con el virus del Zika se debe a que el estudio de su distribución espacial permitiría identificar segmentos de la población con mayor riesgo de padecer la infección, ya que, al contrario que el dengue, la expansión geográfica del virus del Zika afecta a una población que no ha desarrollado inmunidad, lo que aumenta su posible impacto desde la perspectiva de la salud pública (9, 10). Si bien se han desarrollado modelos sobre la dispersión de este flavivirus (11), una de las características de los estudios ya realizados es que adoptaron una perspectiva global y sus hallazgos ponen de manifiesto la necesidad de realizar estudios desde una perspectiva local.

Como ejemplos de estudios que han incorporado la perspectiva geográfica en México cabe destacar uno que describe la situación del virus chikungunya, aunque el análisis se limita al contexto estatal (5), y otros recientes que incorporan un enfoque regional para América Latina (12). Además, la caracterización geográfica de las especies de mosquitos transmisores se ha realizado para zonas costeras en México y ha demostrado que con la incorporación de herramientas de análisis espacial se entienden mejor estos fenómenos de salud pública (13).

Modelizar la distribución geográfica de los egresos hospitalarios por FVPM a escala local es un tema que todavía queda por resolver y que permitiría monitorizar el efecto de la enfermedad a largo plazo, ya que, tal como ha documentado la Organización Mundial de la Salud, también existe una asociación entre la infección por virus del Zika y el desarrollo de malformaciones fetales, incluida la microcefalia: “Basado en la investigación disponible a la fecha, existe un consenso científico que indica que el virus del Zika es una causa de microcefalia y de síndrome de Guillain-Barré” (14).

El objetivo principal de este estudio es conocer la estructura espacial de los egresos hospitalarios de pacientes con FVPM en México y caracterizar su evolución histórica en el periodo 2004-2014.

Además, para entender mejor la diseminación de este fenómeno de salud pública, se pretende averiguar si existe autocorrelación espacial entre los egresos hospitalarios de pacientes con FVPM empleando métodos estadísticos formales a fin de establecer áreas de influencia a escala municipal y detectar los patrones espaciales en el contexto de los procesos que generan dicha configuración. El término autocorrelación espacial se define como la existencia de asociación estadística entre observaciones distribuidas a lo largo de un espacio geográfico determinado.

Por último, para caracterizar las zonas de riesgo a escala local por la presencia del vector trasmisor en las inmediaciones de asentamientos humanos, se analizan explícitamente los determinantes ambientales asociados con la presencia del mosquito *Aedes aegypti*, como la altitud y la temperatura.

## MATERIALES Y MÉTODOS

Se realizó un análisis exploratorio e inferencial de corte transversal considerando como unidad de análisis la demarcación estatal y la municipal. La fuente primaria de datos fue la de los registros de egresos hospitalarios del Sistema Nacional de Información en Salud (SINAIS) del periodo 2004-2014. Para poder comparar los resultados en el contexto internacional, los casos de FVPM se identificaron con los siguientes códigos de la CIE-10: fiebre del dengue (dengue clásico) (A90X), fiebre del dengue hemorrágico (A91X), enfermedad por virus chikungunya (A920), otras fiebres víricas transmitidas por mosquitos (A92), fiebre vírica transmitida por mosquito sin otra especificación (A929), y otras fiebres víricas especificadas transmitidas por mosquitos (A928).

Como fuente de información geográfica se utilizó la incluida en la cartografía digital de elevaciones para México generada por el Instituto Nacional de Estadística y Geografía (INEGI) a resolución de 15 metros. Ésta es un conjunto de datos que representa las elevaciones del territorio continental mexicano mediante valores que indican puntos sobre la superficie del terreno cuya ubicación geográfica se encuentra definida por coordenadas (X e Y) a las cuales se asignan valores que representan las elevaciones (Z) (15). Los datos de la temperatura media anual los proporcionó el Instituto de Geografía de la Universidad Nacional Autónoma de México. Con estos datos, que digitaliza la Comisión Nacional para el Conocimiento y Uso de la Biodiversidad (CONABIO), se puede representar el perímetro de los polígonos asociados con un rango de temperaturas medias en el territorio nacional a partir de información estadística de 1 800 estaciones del Sistema de Observación Climatológica del país, que abarca el período de 1921 a 1980 (16).

Como la estructura demográfica tiene influencia sobre la cantidad nominal de casos observados en un área geográfica determinada, es importante estimar una medida relativa de concentración. Para medir la concentración geográfica de casos de FVPM a partir de la información sobre egresos hospitalarios a nivel estatal, se estimó el cociente de localización (LQ), un indicador que por su potencial para detectar patrones de comportamiento espaciales se ha utilizado en el campo de bioestadística y en estudios empíricos epidemiológicos (17-19). Este indicador se estimó con la siguiente fórmula:

$$\text{LQ}={\left(\frac{FVPM_{i}}{\sum_{Z99}^{A00}E}\right)}_{S}/{\left(\frac{\sum_{i=1}^{32}FVPM_{i}}{\sum_{Z99}^{A00}E}\right)}_{N}$$

donde el subíndice *S* corresponde al i-ésimo estado del país y el subíndice N, a la información a nivel nacional. El LQ es el cociente entre la incidencia observada en un espacio geográfico determinado y la incidencia observada en un espacio geográfico base ajustada por el tamaño de su población. En este caso, se contrasta con cada uno de los 32 estados del país. Dos umbrales del índice se consideran como referencia: LQ > 1 indica una concentración superior del padecimiento estudiado para el i-ésimo estado respecto al nivel observado en el contexto nacional y LQ < 1, una concentración menor en el estado respecto al país.

En segundo lugar se estimó la trayectoria histórica de crecimiento de los casos de FVPM en los principales estados con presencia de estas afecciones respecto al nivel nacional en el periodo comprendido entre 2004 y 2014. Como está asociada con los patrones de crecimiento observados a nivel estatal, el análisis de la estructura de edad en la cual se distribuyen los casos de FVPM puede aportar información adicional para conocer el grado de vulnerabilidad de la población a escala local. Para analizar este aspecto, se construyeron curvas de distribución por edad de los casos ocurridos mediante la función (curvas) de densidad acumulada de Kernel.

Para complementar el análisis de la estructura espacial con información a escala municipal, se estimaron indicadores locales de autocorrelación espacial (LISA). Este enfoque metodológico se ha aplicado en estudios epidemiológicos para modelizar la transmisión de enfermedades víricas entre otras enfermedades (20). Se trata de una medida de la relación que guarda una variable con el espacio al contrastar la heterogeneidad entre observaciones de interés a medida que se incluye la distancia que hay entre ellas (21). Su supuesto de partida es que a distancias menores se observará mayor similitud en la manifestación del fenómeno estudiado. Para ello, se calculó el índice de autocorrelación espacial Moran I, que permite detectar la existencia de patrones espaciales y si estos son estadísticamente significativos. Con este índice se identifican cuatro clases de patrones según la naturaleza de la asociación de la variable de interés en el territorio, en este caso el número de egresos hospitalarios por FVPM por municipio.

El primer grupo lo integran aquellos municipios que, con un número de casos elevado, se encuentran rodeados por municipios donde dicha magnitud también es alta. Esta clase se denomina cluster alto-alto. La segunda clase incluye aquellos municipios con un número de casos bajo que se encuentran rodeados por municipios también con pocos casos (se denomina cluster bajo-bajo). Siguiendo este orden, las dos clases restantes posibles son: cluster alto-bajo y cluster bajo-alto. La primera contiene municipios con un número alto de casos que están rodeados por municipios con pocos casos y la segunda, municipios con pocos casos rodeados por municipos con muchos.

Por último, para poder hacer comparaciones a escala local, se estimaron las tasas de morbilidad ajustadas por edad y sexo a nivel nacional y estatal.

## RESULTADOS

**FIGURA 1.**
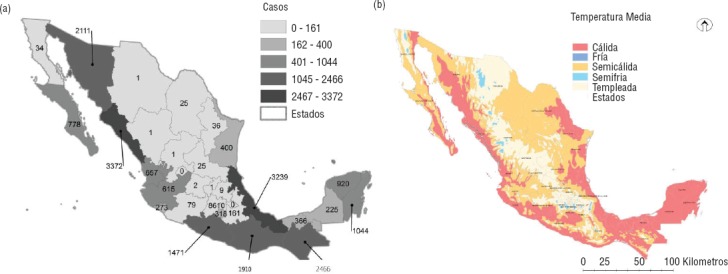
a) Casos de fiebre vírica por picadura de mosquito por estado, 2014 y b) temperatura media anual, México, 2014

El mapa a) de la figura 1 muestra el número de casos de FVPM por estado en 2014, y el mapa b), la regionalización climática, una variable contextual importante, porque permite contrastar la correspondencia entre los casos de FVPM y la presencia del vector transmisor del virus (hay estudios experimentales que indican que la especie del mosquito *Aedes aegypti* afronta serias dificultades para sobrevivir a temperaturas inferiores a los 14-15 °C) (22). Los cinco estados con mayor incidencia en 2014 fueron Sinaloa, Veracruz, Chiapas, Sonora y Oaxaca.

***Fuente:*** Elaboración propia a partir de los datos del Sistema Nacional de Información en Salud (SINAIS) sobre los egresos hospitalarios en 2014 y de las temperaturas de la Comisión Nacional para el Conocimiento y Uso de la Biodiversidad (CONABIO). El mapa b) contiene la información estadística de 1 800 estaciones del sistema de observación climatológica en el país y abarca el período de 1921 a 1980. Los intervalos de referencia son: temperatura cálida con temperatura media de 22 °C a 26 °C; semicálida con temperatura media de 18 °C a 22 °C; templada, con temperatura media de 12° a 18 °C, semifría, con temperatura media de 5 °C a 12 °C, y fría, con temperatura media de 5 °C.

**FIGURA 2.**
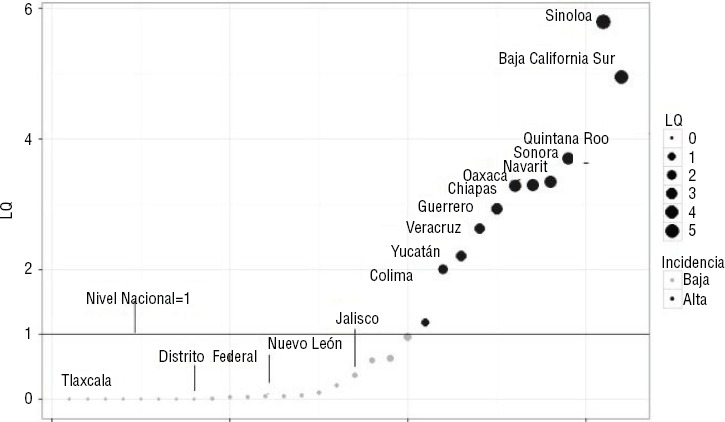
Cociente de localización de los casos de fiebre vírica por picadura de mosquito por estado, México, 2014

Los LQ se presentan en la figura 2. Cuando se ajusta por el tamaño población en cada estado, el escenario de concentración se modifica y, si bien Sinaloa continua siendo el estado con la incidencia más alta, con un nivel 6 veces superior al contexto nacional, los estados que bajo el enfoque de medición nominal parecían de alta incidencia en realidad tienen una concentración relativa menor y ya no se encuentran entre los cinco de mayor incidencia. El caso de estados como Oaxaca y Veracruz ilustra este punto. Por el contrario, los estados de Baja California Sur, Quintana Roo y Nayarit, de hecho, se ubican entre los cinco estados con mayor incidencia en el país con un LQ de 4,95, 3,7 y 3,3 respectivamente, lo que muestra una alta incidencia con una concentración de casos equivalente a cinco veces el escenario nacional para el caso de Quintana Roo y un escenario que triplica el nivel observado en México en el caso de Nayarit.

*Fuente:* Elaboración propia con datos del Sistema Nacional de Información en Salud (SINAIS) sobre los egresos hospitalarios en 2014.

**FIGURA 3.**
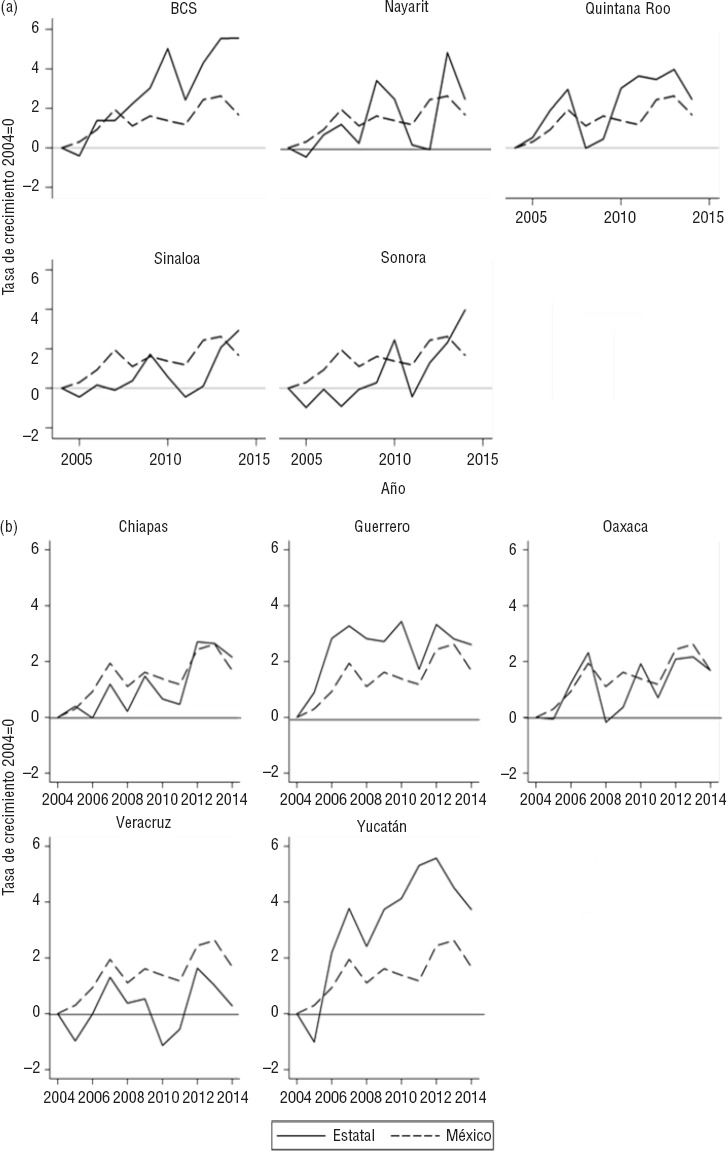
Tasa de crecimiento de los casos de fiebre vírica por picadura de mosquito en los estados con mayor concentración, México, 2004–2014

Para analizar la evolución de casos de FVPM en los cinco estados con mayor concentración, se estimaron sus tasas de crecimiento durante la última década respecto al crecimiento observado para el ámbito nacional, una perspectiva histórica que aporta información sobre el control epidemiológico que se ha realizado en los estados identificados. Las tasas de crecimiento estimadas toman como base el número de egresos hospitalarios de 2004, de tal forma que es posible evaluar la evolución histórica de este padecimiento. Para mayor referencia se estimó la tasa de crecimiento nacional. Este análisis permite distinguir dos grupos: el primero (figura 3, mapa a)) contiene los cinco estados con mayor LQ de todo el país, y en él destaca, por su ritmo de crecimiento, el estado de Baja California Sur, con un importante crecimiento que se hace evidente por la brecha respecto a la trayectoria observada en el contexto nacional para el periodo. En el segundo grupo (figura 3, mapa b)) sobresale el hecho de que está conformado por estados localizados en el sureste del país, donde la persistencia de elevados niveles de pobreza constituye un rasgo de interés para la estrategia de atención de los casos de FVPM y del manejo de las complicaciones asociadas. En este grupo cobra relevancia la tendencia ascendente observada en Yucatán, donde el número casos ha crecido a un ritmo cuatro veces mayor que el patrón observado en el resto del país durante la última década. Asimismo, en los estados se identificó la formación de clusters entre municipios.

**FIGURA 4.**
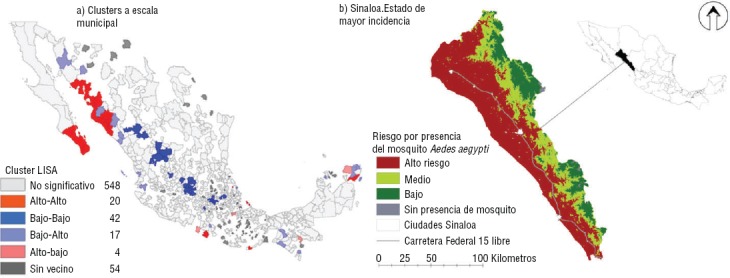
a) Detección de clusters a escala municipal de egresos hospitalarios por casos de fiebre vírica por picadura de mosquito mediante el índice local de autocorrelación espacial Moran I. b) Estado con el mayor número de egresos hospitalarios de dichos casos, México, 2014

El mapa de la figura 4 muestra los clusters identificados a escala municipal junto con el nivel de riesgo de infección por la presencia del vector transmisor según la variable contextual altitud para Sinaloa, el estado con la mayor concentración de casos de FVPM en México. Es posible confirmar que este fenómeno tiene un comportamiento focalizado. La estimación muestra asimismo un patrón que afecta notablemente al estado de Sinaloa en el norte, donde, según la variable contextual elevación, se puede apreciar que sus principales áreas pobladas se encuentran en zonas de alto riesgo por la presencia del vector transmisor, ya que según se ha documentado la especie *Aedes aegypti* tiene una capacidad limitada para sobrevivir a una altitud mayor de 2 000 metros sobre el nivel del mar (23, 24).

También se observa la presencia del vector en conjuntos de municipios muy específicos en el sur del país (Acapulco, Santa María Huatulco o el municipio de Solidaridad en el estado de Quintana Roo), no sólo en la península de Yucatán, donde el caso de Mérida es notable. En total, se detecta alta concentración en clusters integrados por 20 municipios de los cuales 9 se localizan en el estado de Sinaloa.

**FIGURA 5.**
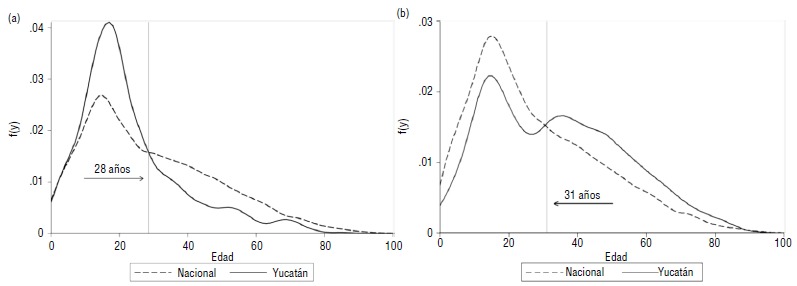
Curvas de densidad. Distribución de la edad de los egresos hospitalarios de casos de fiebre vírica por picadura de mosquito en a) Yucatán y b) Sinaloa, México, 2014

En la figura 5 aparecen las curvas de densidad acumulada de Kernel, que indican el comportamiento para Sinaloa y Yucatán, estados de importancia en la región norte y sur, respectivamente. En ambos estados se aprecia una marcada diferencia en la estructura de edad de la población afectada respecto a la del país. En Sinaloa, la incidencia es mayor en las personas entre 31 y 60 años de edad, mientras que en Yucatán la incidencia se acentúa en la población menor de 28 años. Como estas diferencias en la estructura de edad exigen estimar indicadores para controlar el efecto de posibles sesgos asociados con las características demográficas locales, se estimaron las tasas de morbilidad por FVPM ajustadas por edad a nivel nacional y para los 10 estados con mayor concentración de casos del país. En México, estas tasas en hombres y mujeres fueron, respectivamente, 17 casos por 100 000 habitantes y 18 casos por 100 000. En los estados de Sinaloa, Baja California Sur, Sonora y Nayarit del norte del país las tasas fueron 110, 88, 64, 53 por 100 000 en los hombres y 117, 124, 82 y 56 por 100 000 en las mujeres, respectivamente, y en los cinco estados del sur con mayor morbilidad (Quintana Roo, Chiapas, Oaxaca, Veracruz y Yucatán), 68, 41, 51, 48 y 42 en los hombres y 67, 49, 45, 42 y 41 en las mujeres.

Los estados con tasas de morbilidad igual a cero, es decir, libres de casos de FVPM, forman un patrón que se extiende por la zona central del país desde el Distrito Federal, el estado de México y Tlaxcala, e incluye los estados de Querétaro, Guanajuato, Aguascalientes, Zacatecas, Durango y Chihuahua.

*Fuente:* Elaboración propia con datos del Sistema Nacional de Información en Salud (SINAIS) sobre los egresos hospitalarios en 2014. El grupo a) contiene los estados con mayor concentración detectados según el orden descendente del cociente de localización y el b), el segundo grupo de mayor concentración.

## DISCUSIÓN

Dado el vínculo entre el vector transmisor común de las enfermedades incluidas en el término FVPM y las infecciones emergentes como el zika, conocer la distribución espacial de dicho vector constituye una fuente de información importante para diseñar estrategias de control de estas enfermedades. En relación con su control en México, se ha comprobado que el subdiagnóstico es un rasgo históricamente característico en el país (5). Los resultados del presente estudio indican la existencia de clusters específicos de casos de FVPM y, por ello, la focalización de medidas de control en los espacios detectados puede contribuir al uso eficiente de recursos designados para estrategias preventivas.

Más aún, la ubicación de los clusters en los estados de Oaxaca, Guerrero, Chiapas y Veracruz concuerdan con la hipótesis de estudios recientes sobre el vínculo entre la expansión de enfermedades por flavivirus y las condiciones de pobreza (25), lo cual pone de manifiesto la necesidad de desplegar programas integrales de intervención que incidan en estas condiciones.

Un segundo elemento derivado de los resultados de este estudio es la posibilidad de disponer de una base de datos de referencia para futuras investigaciones sobre los efectos sociales a largo plazo de la FVPM. En este sentido cobran especial relevancia la relación de estas enfermedades con el desarrollo de neuropatías como el síndrome de Guillain-Barré, así como sus efectos en las condiciones de la salud maternoinfantil, un área de investigación en la cual está trabajando la comunidad científica mundial ante los efectos neuroteratogénicos vinculados con el virus del Zika (26).

A este respecto, las elevadas tasas de morbilidad encontradas en la población de mujeres de los estados de Sinaloa y Baja California Sur, con 117 y 124 egresos por 100 000 habitantes, respectivamente, equivalentes a seis veces las tasas del país, dan cuenta de la importancia de obtener más información sobre la distribución espacial de la población afectada y de reforzar los mecanismos de monitorización epidemiológica regional.

Además, los resultados han permitido delimitar las zonas de alta, baja y nula incidencia, y un corredor libre de casos de FVPM. Esta información reviste interés en el contexto de los mecanismos de trasmisión identificados del virus del Zika, que incluyen el contacto sexual, motivo por el cual es importante mantener un seguimiento de cerca de la evolución de casos en el corredor identificado.

***Fuente:*** Elaboración propia con datos del Sistema Nacional de Información en Salud (SINAIS) sobre los egresos hospitalarios en 2014, de la Comisión Nacional para el Conocimiento y Uso de la Biodiversidad (CONABIO) sobre temperatura y del Modelo Mexicano de Elevaciones. Las elevaciones se miden en metros sobre el nivel medio del mar (msnm) con esta escala ordinal de riesgo: alto riesgo: 229, medio 229-651, bajo: 652-2000, sin presencia del mosquito > 2000 msnm.

***Fuente:*** Elaboración propia con datos del Sistema Nacional de Información en Salud (SINAIS) sobre los egresos hospitalarios en 2014.

En conclusión, en el presente estudio se han identificado 20 municipios que integran clusters de alta incidencia de casos de FVPM y se han estimado los niveles de concentración relativa del número de casos por estado, que muestran su correspondencia con variables contextuales identificadas en estudios precedentes como la temperatura ambiental y la altitud. Además, se ha observado un vínculo entre las zonas de alto riesgo por la presencia del vector transmisor y condiciones sociales como la pobreza, cuyo conocimiento es sumanente útil para diseñar estrategias de prevención y control en México de estas infecciones víricas emergentes transmitidas por artrópodos, como la del virus Zika.

### Agradecimiento.

El autor agradece al Consejo Nacional de Ciencia y Tecnología el acceso a la infraestructura disponible a través del sistema Nacional de investigadores.

### Financiación.

Este estudio no ha recibido financiación.

### Declaración.

Las opiniones expresadas por los autores son de su exclusiva responsabilidad y no reflejan necesariamente los criterios ni la política de la Organización Panamericana de la Salud o de la RPSP/PAJPH.
